# Massive thymoma of the mid-posterior mediastinum: an unprecedented case in a young adult*

**DOI:** 10.1590/0100-3984.2014.0025

**Published:** 2016

**Authors:** Karen Fernandes de Oliveira, Marcio Maciel Rodrigues, Gesner Pereira Lopes, Renan Sandoval de Almeida, Juliana Lopes Lusvarghi, João Paulo Vieira dos Santos

**Affiliations:** 1 MD, Resident in Dermatology at the Hospital Universitário de Brasília (HUB), Brasília, DF, Brazil.; 2 Fellow in Oncological Radiology at the Instituto do Câncer do Estado de São Paulo Octavio Frias de Oliveira (Icesp), São Paulo, SP, Brazil.; 3 Head of the Department of Radiology and Diagnostic Imaging at the Universidade Federal do Triângulo Mineiro (UFTM), Uberaba, MG, Brazil.; 4 MD, Resident in Clinical Medicine at the Universidade Federal de Uberlândia (UFU), Uberlândia, MG, Brazil.; 5 MD, Resident in Plastic Surgery at the Hospital Heliópolis, São Paulo, SP, Brazil.; 6 Thoracic Surgeon at the Hospital de Clínicas da Universidade Federal do Triângulo Mineiro (UFTM), Uberaba, MG, Brazil.

**Keywords:** Thymoma, Middle mediastinum, Posterior mediastinum, Ectopic tumor

## Abstract

We report an unprecedented case of ectopic thymoma in a young adult. A 33-year-old male presented with a 10-day history of non-productive cough and fever. Investigation revealed mediastinal widening without pulmonary involvement. Computed tomography showed a large mass-14.8 × 10.8 × 8.4 cm-in the mid-posterior mediastinum, and a biopsy obtained by video-assisted thoracoscopy indicated that the mass was a tumor. Immunohistochemistry showed combined thymoma type AB1. Because of the considerable proportions of the tumor and its close proximity to major structures, the patient was treated with chemotherapy.

## Abstract (pt)

Apresentamos caso inédito de volumoso timoma ectópico em adulto jovem. Homem de 33 anos de idade, encaminhado com tosse seca e febre diária havia 10 dias. Durante investigação observou-se alargamento mediastinal sem comprometimento pulmonar. Um tumor mediastinal medioposterior, medindo 14,8 × 10,8 × 8,4 cm, foi diagnosticado após tomografia computadorizada e biópsia videotoracoscópica. A imuno-histoquímica revelou timoma misto AB1. Devido à íntima relação com estruturas nobres e grandes proporções, optou-se pela quimioterapia.

## INTRODUCTION

Although thymoma is the most common primary tumor of the anterior mediastinum, it accounts for less than 1% of all neoplasms in adults^(1,2)^. The involvement of middle and posterior mediastinum is rare, only 16 cases of thymoma in the middle mediastinum having been reported^(2-10)^. The incidence of thymoma peaks between 50 and 60 years of age. Here, we describe the challenge of diagnosing this rare neoplasm in a mildly symptomatic young adult.

## CASE REPORT

**Figure 1:**
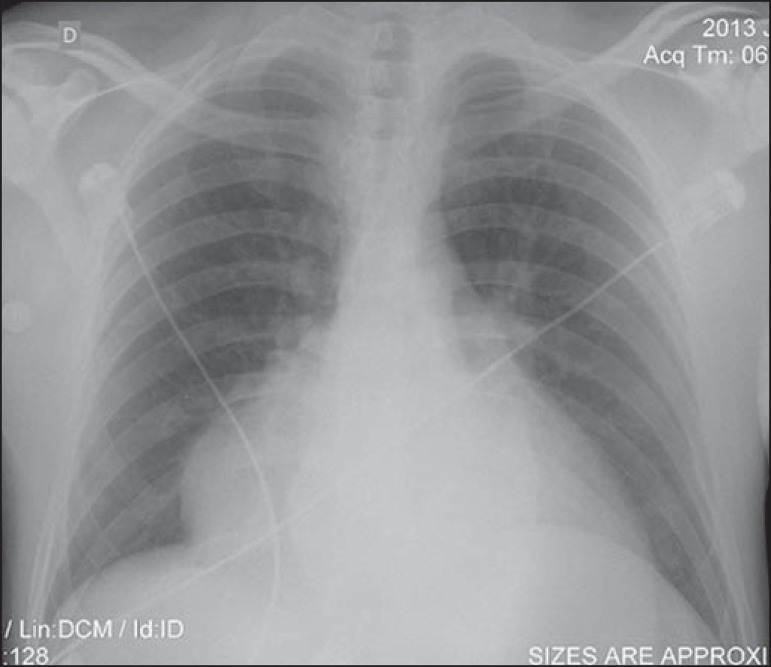
Chest X-ray showing a mass with well-defined borders. Pulmonary parenchyma with preserved transparency.

**Figure 2:**
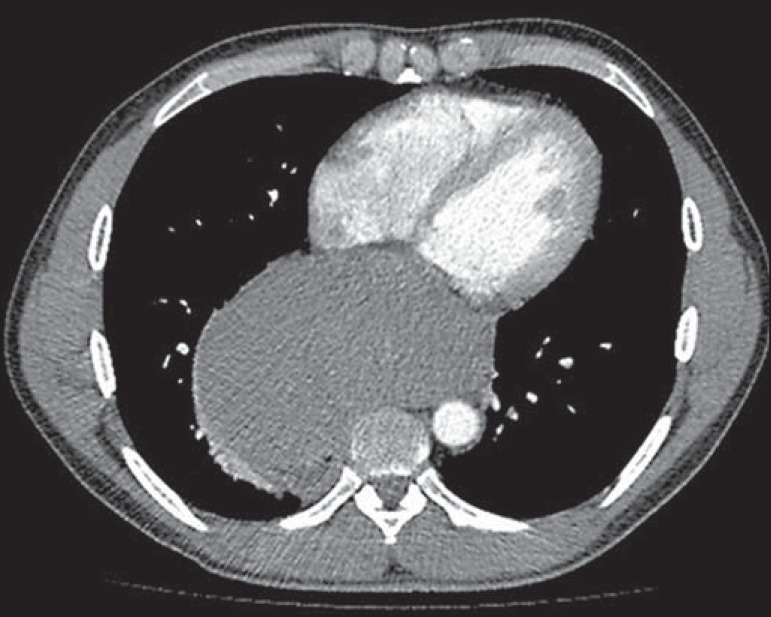
Contrast-enhanced CT of the chest, with coronal (**A**) and sagittal (**B**) reconstructions, showing a mass with a heterogeneous texture and discrete heterogeneous contrast enhancement, measuring 10.8 × 8.4 × 14.8 cm, in the mid-posterior mediastinum. The mass presents an intimate relationship with the great mediastinal vessels, right main bronchus, vertebral bodies, right atrium, diaphragm, and esophagus, albeit without direct signs of invasion of any of those structures.

**Figure 3:**
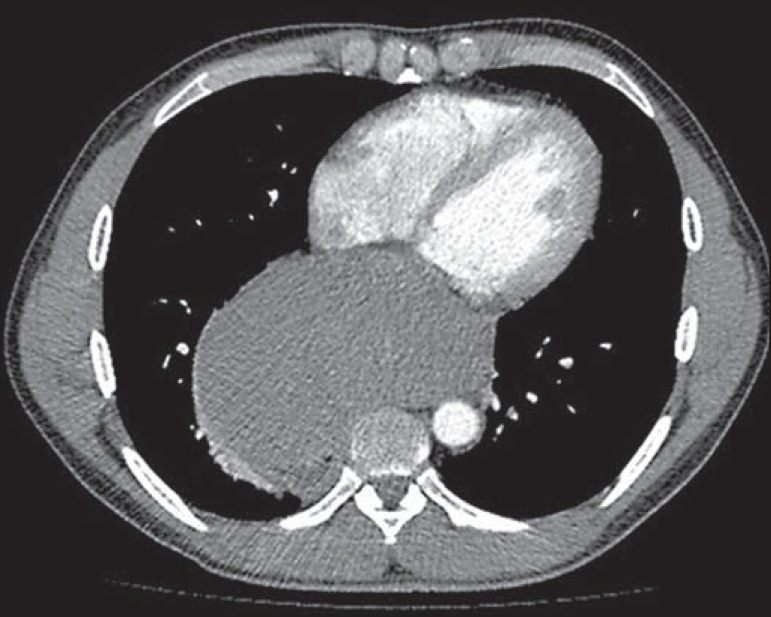
Axial contrast-enhanced CT of the chest showing a mass in the mid-posterior mediastinum.

A 33-year-old man was hospitalized in July of 2013 to investigate a 10-day history of dry cough and fever. At admission, the physical examination showed no alterations. Laboratory tests showed normocytic, normochromic anemia (Hb = 9.7 g/dL); inversion of the albumin/globulin ratio (A/G ratio = 0.65); a C-reactive protein level of 33.1 mg/dL; thrombocytosis (platelet count = 592,000/mm^3^); and leukocytosis without a shift (leukocyte count = 14,290/mm^3^). The patient evolved to daily fever spikes and tachycardia. A chest X-ray revealed mediastinal widening (Figure 1), and echocardiography was therefore required. The echocardiography showed extrinsic compression of the left atrium by a mediastinal mass, with an ejection fraction of 56%. Computed tomography (CT) of the chest (Figures 2 and 3) showed that the mass measured 14.8 cm at its greatest diameter and was located in the mid-posterior mediastinum. Upper gastrointestinal endoscopy identified extrinsic compression of the distal esophagus and gastric cardia. Barium swallow allowed us to visualize a significant delay in emptying, and protein electrophoresis showed a polyclonal increase in gamma globulins. The main diagnostic hypotheses were lymphoma, giant leiomyoma of the esophagus, neurogenic tumor, and plasmacytoma. Empirical antibiotic therapy was started and resulted in clinical improvement.

For diagnostic clarification, a video-assisted thoracoscopic biopsy was performed. Frozen section analysis of the biopsy material was suggestive of lymphoma. However, immunohistochemistry revealed thymoma type AB1.

The patient was discharged with a referral for outpatient chemotherapy. A follow-up CT scan showed partial regression of the tumor. When the CT scan was reevaluated by the thoracic surgery team in February of 2014, the tumor was still considered unresectable.

## DISCUSSION

Half of all mediastinal tumors-including thymoma, germ cell tumors, thyroid diseases, and lymphoma-have an anterior origin. In the middle mediastinum, congenital cysts prevail, whereas neurogenic tumors prevail in the posterior mediastinum^(1,2,11,12)^.

The thymus is a lymphoid organ that plays a critical role in the maturation of lymphocytes and in cellular immunity. Embryologically, it originates from the third and fourth pharyngeal pouches. During their migration, thymic tissue fragments or accessory lobes can erroneously locate to the cervical region (in 4% of cases) or to the middle mediastinum^(1,7,11,12)^. Including the case presented here, only 17 cases of mediastinal thymoma have been reported. Among those cases, ours is the only one in which the tumor was unresectable, and the individual affected in our cases was younger than those affected in the other cases reported, underscoring the rarity of the case presented here.

The annual incidence of thymoma is 0.15 cases/100,000 population, with no difference between genders, and its incidence increases in adulthood, peaking at 50-60 years of age^(2)^. Most patients are asymptomatic, being diagnosed on the basis of incidental findings on imaging studies. Approximately 40% of symptomatic patients present with myasthenia gravis, the paraneoplastic syndrome most often associated with thymoma^(8,9)^. Our patient reported having a normal diet and showing no signs of dysphagia.

Thymomas originate from the epithelial cells of the thymus. Histologically, the thymus has two regions: the cortex, which is rich in lymphocytes, and the medulla, which is composed of epithelial cells. The histological classification is based on the morphology of the epithelial cells and the proportional relationship between those cells and lymphocytes. Most of the thymomas that are determined to be type A, AB, or B1 have a benign course. Thymoma types B2 and B3 are considered to be malignant, with metastatic potential^(13)^. The most widely used staging system is that devised by Masaoka et al.^(14)^, which involves postoperative pathological evaluation of capsular invasion of the thymus.

Because the tumor could not be resected, radiology played a key role in the case presented here. The initial diagnosis and staging were based on imaging studies, with an emphasis on the detection of locoregional or distant invasion. Approximately 45-80% of thymomas are visible on conventional chest X-rays, which initiated the diagnostic investigation in our patient. For the evaluation of mediastinal masses, CT is the tool of choice^(2,15)^.

Complete surgical resection is the main therapy for invasive and noninvasive thymomas, being the most important predictor of long-term survival. However, radiation therapy and chemotherapy, alone or in combination, produce favorable results, increasing survival and improving the prognosis^(12,16)^.

Thymoma can mimic a variety of diseases, including those with compressive symptoms and paraneoplastic diseases, as well as mediastinal widening. Hence the importance of this case-to expand diagnostic reasoning for a tumor in the middle mediastinum, because it is a rare differential diagnosis to be considered, especially in young patients.
